# The Role of Compulsive Dominance in the Emergence of Paranoid Ideation in Obsessive-Compulsive Disorder

**DOI:** 10.1192/j.eurpsy.2026.11596

**Published:** 2026-07-31

**Authors:** H. Yildiz

**Affiliations:** psychiatry, Turkish Psychiatric Association, Ankara, Türkiye

## Abstract

**Introduction:**

Obsessive-compulsive disorder (OCD) is a chronic psychiatric disorder characterized by obsessions and compulsions. The boundary between OCD and psychotic disorders has been debated, with certain subtypes demonstrating overlapping features. In cases where compulsions are excessively dominant, the persistent reinforcement of perceived threats may create fertile ground for paranoid ideation. This paper examines the contribution of compulsive dominance to paranoid cognition through neurobiological, cognitive, and psychodynamic perspectives, and discusses clinical observations and therapeutic strategies.

**Objectives:**

This article explores how excessive dominance of compulsive behaviors in obsessive-compulsive disorder (OCD) may contribute to the development of paranoid ideation.

**Methods:**

Through literature review and clinical observations, the cognitive, neurobiological, and psychodynamic mechanisms linking compulsive symptoms with paranoia are examined.

**Results:**

Compulsive dominance in OCD may evolve into paranoid ideation, representing a distinct clinical subgroup. Serotonergic deficits, dopaminergic hyperactivity, CSTC dysfunction, and impaired reality testing all contribute to this transformation. Clinical observations support the progression from compulsive behaviors to paranoid beliefs.

**Conclusions:**

In conclusion, compulsive-dominant OCD with paranoia requires tailored interventions. Pharmacological management should combine SSRIs with antipsychotic augmentation, while psychotherapeutic strategies must address both compulsive rituals and paranoid schemas within an integrated framework.

**Disclosure of Interest:**

None Declared

